# Supplementary material for: PD-1 suppresses TCR-CD8 cooperativity during T-cell antigen recognition
Source: Nat Commun. 2021 May 12;12:2746. doi: 10.1038/s41467-021-22965-9 (PMC8115078; doi:10.1038/s41467-021-22965-9)
Supplement: Supplementary file 1 — Supplementary Information [file 41467_2021_22965_MOESM1_ESM.pdf]

Supplementary Fig. 1

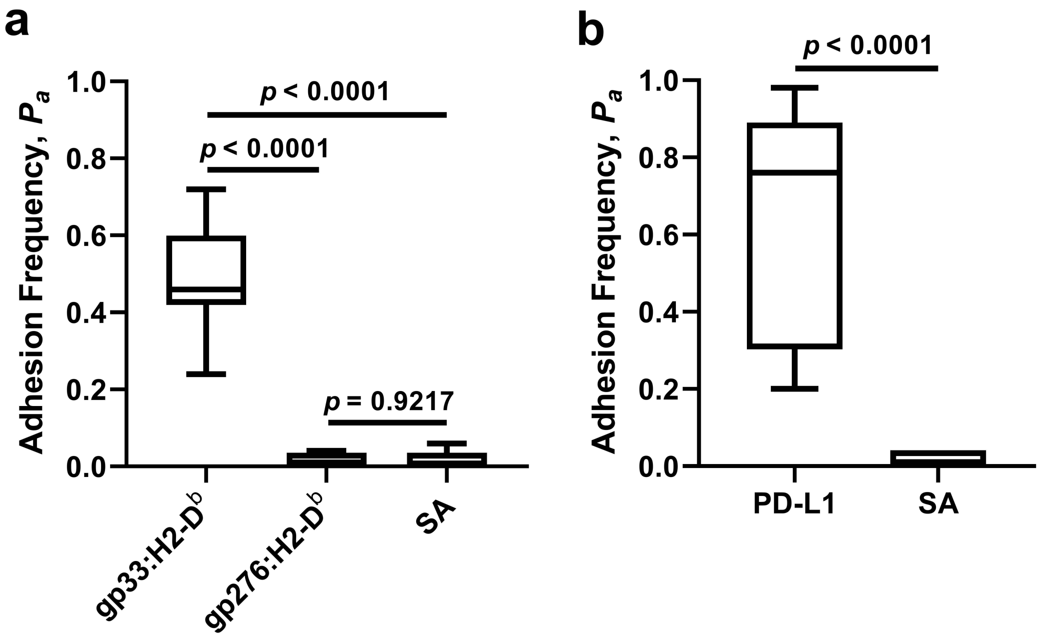

Supplementary Fig. 1 Negative control of 2D kinetic analysis of P14 $CD8^+$ T cells, Related to Fig. 2. a, Adhesion frequency (P_a) of *in vitro* activated P14 $CD8^+$ T cells binding to RBCs of the same biotinylation level coated with gp33:H2-D^b, gp276:H2-D^b or SA at 5-s contact time (n = 12, 8, and 8 cells). **b,** Adhesion frequency (P_a) of *in vitro* activated P14 $CD8_+$ T cells binding to RBCs of the same biotinylation level coated with PD-L1 or SA at 5-s contact time (n = 13 and 8 cells). Data are presented by the box-whisker plots with the center line labeling median, the box containing the two middle quantiles and the whiskers marking the min and the max. *p* values were calculated using Mann-Whitney test.

Supplementary Fig. 2

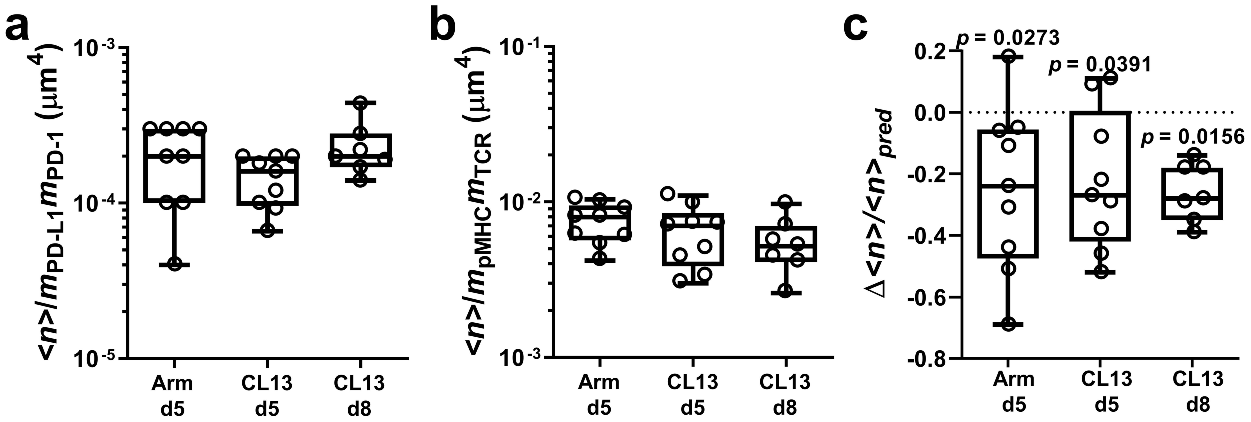

Supplementary Fig. 2 2D kinetic analysis of negative cooperativity using CD8$^+$ T cell from LCMV infected P14 chimeric mice, Related to Fig. 2. P14 chimeric mice were infected with LCMV Armstrong (Arm) or clone 13 (CL13). P14 CD8$^+$ T cells were sorted at indicated days and tested for their binding to RBCs coated with PD-L1, gp33:H2-D^b, or both. **a,b,** Comparisons of normalized numbers of PD-1–PD-L1 (**a**, n = 9, 9, and 7 cells) and TCR–pMHC (**b**, n = 9, 9, and 7 cells) bonds formed between T cells and RBCs bearing PD-L1 or pMHC. **c,** Comparison of normalized bond reductions ($\Delta\langle n\rangle/\langle n\rangle_{pred}$) of indicated T cells interacting with RBCs bearing PD-L1 and gp33:H2-D^b (n = 9, 9, and 7 cells). Data are presented by the box-whisker plots with the center line labeling median, the box containing the two middle quantiles and the whiskers marking the min and the max. The n values indicate the number of T cells analyzed. Not significant unless indicated. *p* values were calculated using one sample Wilcoxon test comparing the median to zero.

Supplementary Fig. 3

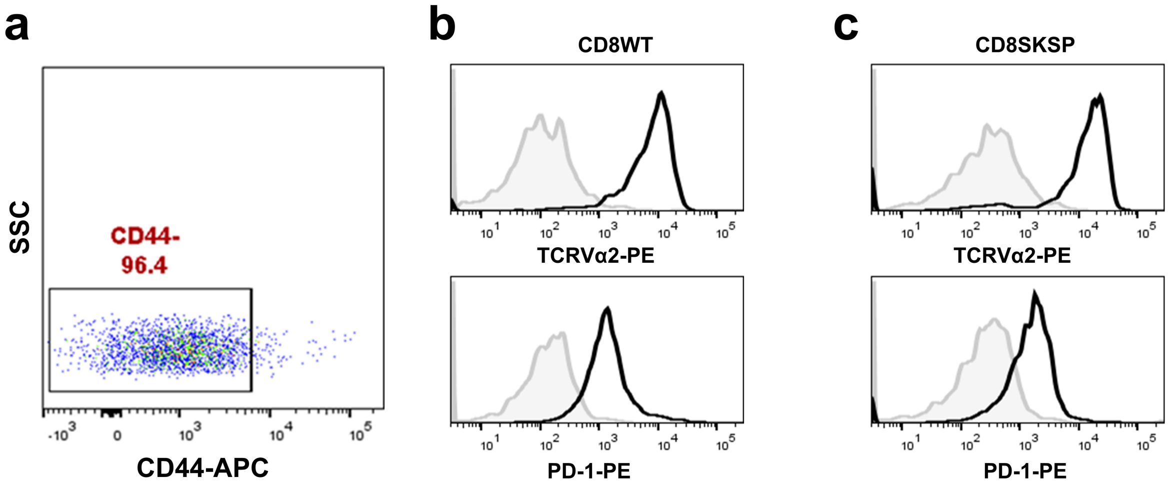

Supplementary Fig. 3 Expression of CD44, TCR, and PD-1 on P14 CD8$^-$CD4$^-$ thymocytes, Related to Fig. 5. a, Representative CD44 vs SSC plot showing most P14 CD8$^-$CD4$^-$ thymocytes analyzed are CD44$^-$ (DN3 and DN4). **b,c,** Representative histogram plots showing similar TCR and PD-1 expression in P14 CD8$^-$CD4$^-$ thymocytes transduced with retrovirus of **b)** CD8WT and PD-1 or **c)** CD8SKSP and PD-1.

Supplementary Fig. 4

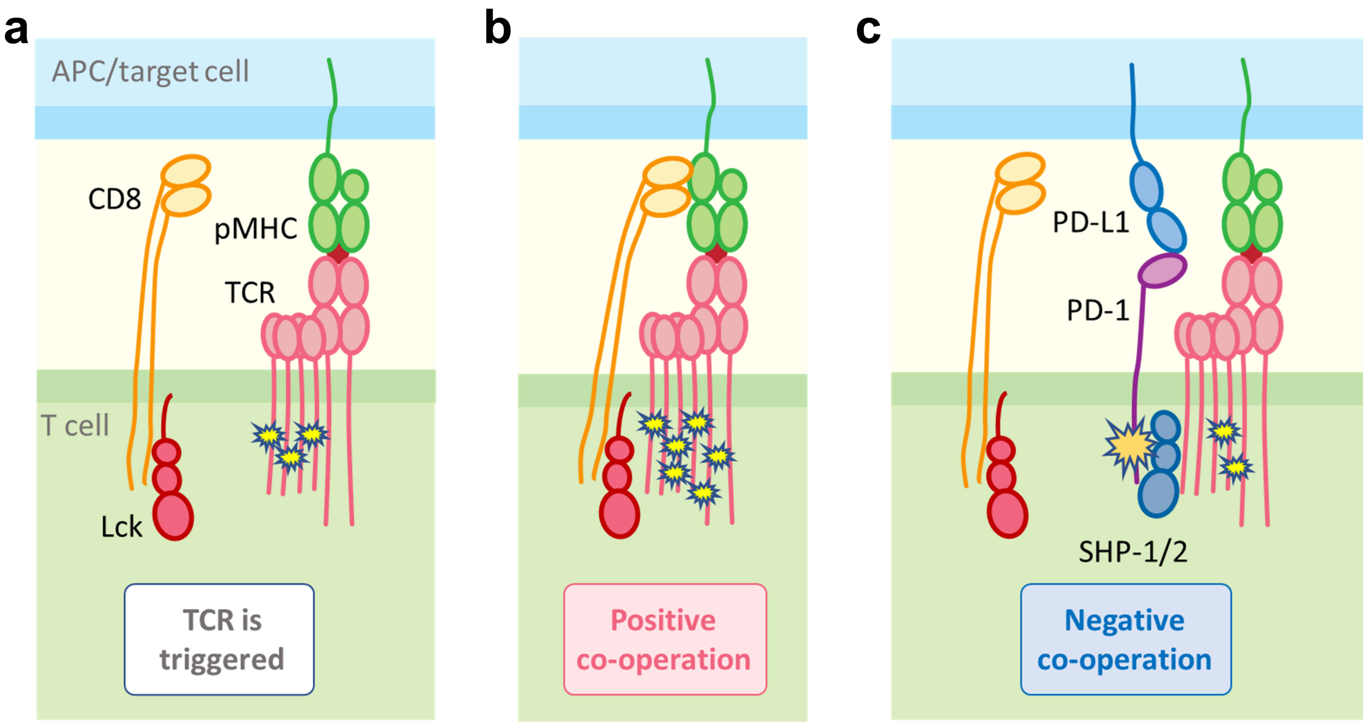

Supplementary Fig. 4 Model for PD-1 inhibition of the initiating steps of T-cell antigen recognition, Related to Figs. 1-6. a, TCR engagement with pMHC triggers phosphorylation of CD3 ITAMs by Lck that is likely not associated with CD8. **b,** CD8-associated Lck binds to phosphorylated CD3 ITAMs (and possibly also phosphorylated ZAP70 bound to the phosphorylated CD3, not shown), recruiting CD8 to TCR-CD3. This enables positive cooperative pMHC binding by the TCR and CD8 to augment TCR-triggered signals. **c,** Concurrent engagement of PD-1 by PD-L1 induces phosphorylation of its ITIM and ITSM, which recruits and activates SHP1/2. Activated SHP1/2 dephosphorylates TCR proximal signaling molecules (e.g., CD3 and ZAP70) and disrupts the TCR-CD8 cooperative binding of pMHC, manifesting a negative cooperativity of the two axes. This counteracts the CD8-augmentation of T-cell antigen recognition and thereby suppresses T-cell activation at the early phase. Note that only new elements supported by the findings in this paper are highlighted here. Elements reported by others that show how PD-1 inhibits downstream signals triggered by TCR and CD28 are not included.

Supplementary Table 1. Summary of sample sizes for bond lifetime measurements in Figure 4.

Figure	Group	Sample size
4F	PD-L1	826
	gp33:H2-D^b	529
	gp33:H2-D^b +Lck In	294
	gp33:H2-D^bα3A2	488
4G	PD-L1	46
	gp33:H2-D^b	88
	gp33:H2-D^b +Lck In	70
	gp33:H2-D^bα3A2	46
4H	measured 3pN	67
	measured 6pN	116
4J	measured 3pN	40
	measured 6pN	42
4L	measured 3pN	54
	measured 6pN	68
