# Supplementary material for: PD-1 suppresses TCR-CD8 cooperativity during T-cell antigen recognition
Source: Nat Commun. 2021 May 12;12:2746. doi: 10.1038/s41467-021-22965-9 (PMC8115078; doi:10.1038/s41467-021-22965-9)
Supplement: Supplementary file 3 — Reporting summary [file 41467_2021_22965_MOESM3_ESM.pdf]

nature research

Corresponding author(s): Cheng Zhu

Last updated by author(s): Mar 22, 2021

Reporting Summary

Nature Research wishes to improve the reproducibility of the work that we publish. This form provides structure for consistency and transparency in reporting. For further information on Nature Research policies, see our Editorial Policies and the Editorial Policy Checklist.

Statistics

For all statistical analyses, confirm that the following items are present in the figure legend, table legend, main text, or Methods section.

n/a	Confirmed	
☐	☒	The exact sample size (*n*) for each experimental group/condition, given as a discrete number and unit of measurement
☐	☒	A statement on whether measurements were taken from distinct samples or whether the same sample was measured repeatedly
☐	☒	The statistical test(s) used AND whether they are one- or two-sided<br>*Only common tests should be described solely by name; describe more complex techniques in the Methods section.*
☒	☐	A description of all covariates tested
☒	☐	A description of any assumptions or corrections, such as tests of normality and adjustment for multiple comparisons
☐	☒	A full description of the statistical parameters including central tendency (e.g. means) or other basic estimates (e.g. regression coefficient) AND variation (e.g. standard deviation) or associated estimates of uncertainty (e.g. confidence intervals)
☐	☒	For null hypothesis testing, the test statistic (e.g. *F*, *t*, *r*) with confidence intervals, effect sizes, degrees of freedom and *P* value noted<br>*Give P values as exact values whenever suitable.*
☒	☐	For Bayesian analysis, information on the choice of priors and Markov chain Monte Carlo settings
☒	☐	For hierarchical and complex designs, identification of the appropriate level for tests and full reporting of outcomes
☒	☐	Estimates of effect sizes (e.g. Cohen's *d*, Pearson's *r*), indicating how they were calculated

Our web collection on statistics for biologists contains articles on many of the points above.

Software and code

Policy information about availability of computer code

Data collection	Volocity 6.3.1, Labview 2016, Micro-Manager 1.4, FACSDiva v9
Data analysis	Labview 2016, Matlab 2020a, ImageJ 1.52, Microsoft Excel 2016, Flowjo v10, GraphPad Prism 8

For manuscripts utilizing custom algorithms or software that are central to the research but not yet described in published literature, software must be made available to editors and reviewers. We strongly encourage code deposition in a community repository (e.g. GitHub). See the Nature Research guidelines for submitting code & software for further information.

Data

Policy information about availability of data

All manuscripts must include a data availability statement. This statement should provide the following information, where applicable:
- Accession codes, unique identifiers, or web links for publicly available datasets
- A list of figures that have associated raw data
- A description of any restrictions on data availability

Data supporting the findings of this study are presented in the article and supplementary materials and are available from the corresponding authors upon reasonable request. Source data are presented for the following figures.
Fig. 1 (b, c, e, g); Fig. 2 (f,i); Fig. 3 (a,b,d,e,f,h); Fig. 4 (a,c,h,j,l); Fig. 5 (c,d,e); Fig. 6(e,f,g); Sup fig. 1; Sup fig. 2.

Field-specific reporting

Please select the one below that is the best fit for your research. If you are not sure, read the appropriate sections before making your selection.

- [x] Life sciences
- [ ] Behavioural & social sciences
- [ ] Ecological, evolutionary & environmental sciences

For a reference copy of the document with all sections, see nature.com/documents/nr-reporting-summary-flat.pdf

Life sciences study design

All studies must disclose on these points even when the disclosure is negative.

Sample size	No Statistical method was used to determine the sample sizes. Sample sizes were determined based on applicable standards in the field that ensure reproducibility and statistical power for comparison among different groups.
Data exclusions	Bond lifetime events (Figures 4f-m) with occasional significant force drift or signatures of multiple bond rupture were excluded.
Replication	Replication were performed 2-3 times independently to ensure reproducibility and increase the sample size (cells or bond lifetime events). Groups with fewer than 10 cells were from single experiment. All attempts at replication were successful.
Randomization	Mice, cells, and all sample preparations were treated following consistent standards and allocated randomly into different groups.
Blinding	Blinding was not applied due to the complexity of sample preparation and experiment procedure. However, all procedures were applied equally to all samples. We also we had independent person other than the experimenter to analyze the data.

Reporting for specific materials, systems and methods

We require information from authors about some types of materials, experimental systems and methods used in many studies. Here, indicate whether each material, system or method listed is relevant to your study. If you are not sure if a list item applies to your research, read the appropriate section before selecting a response.

Materials & experimental systems

n/a	Involved in the study	
☐	☒	Antibodies
☐	☒	Eukaryotic cell lines
☒	☐	Palaeontology and archaeology
☐	☒	Animals and other organisms
☐	☒	Human research participants
☒	☐	Clinical data
☒	☐	Dual use research of concern

Methods

n/a	Involved in the study	
☒	☐	ChIP-seq
☐	☒	Flow cytometry
☒	☐	MRI-based neuroimaging

Antibodies

Antibodies used

PE-conjugated anti-mPD-1 (clone J43, 551892), anti-mTCR Vα2 (clone B20.1, 553289), anti-mCD8α (clone 53-6.7, 553033), isotype RatIgG2a,λ (clone B39-4, 557076), isotype RatIgG2a,κ (clone A95-1, 553989), and isotype american hamster IgG2,κ (clone B81-3, 550085) were from BD Pharmingen.
PE-conjugated anti-mPD-L1 (clone MIH5, 12-5982-82) and biotinylated anti-human IgG1 (clone HP6070, MH1515) were from ThermoFisher Scientific.
BV421-conjugated anti-CD4(clone GK1.5, 100438), PE-conjugated anti-H2-Db (clone KH95, 111508), anti-mCD8α (clone 53-6.7, 100708), and isotype ratIgG1,κ (clone RTK2071, 400408) were from Biolegend.
APC-conjugated anti-CD44 (clone IM7, 20-0441-U100) was from Tonbo biosciences.
Biotinylated anti-His tag (cat. 34440) was from Qiagen.

Validation

PE-conjugated anti-mPD-1 (clone J43), BD Pharmingen, Catalog No. 551892
Agata Y, Kawasaki A, Nishimura H, et al. Expression of the PD-1 antigen on the surface of stimulated mouse T and B lymphocytes. Int Immunol. 1996 May; 8(5):765-772.
Ansari MJ, Salama AD, Chitnis T, et al. The programmed death-1 (PD-1) pathway regulates autoimmune diabetes in nonobese diabetic (NOD) mice. J Exp Med. 2003 July; 198(1):63-69.
In Supplementary Figures 3b,c, DN thymocytes from P14 transgenic mice expressing PD-1 were positively stained with this antibody.

PE-conjugated anti-mTCR Vα2 (clone B20.1), BD Pharmingen, Catalog No. 553289
Grégoire C, Rebaï N, Schweisguth F, et al. Engineered secreted T-cell receptor alpha beta heterodimers.. Proc Natl Acad Sci USA. 1991; 88(18):8077-81.
Pircher H, Rebaï N, Groettrup M, et al. Preferential positive selection of V alpha 2+ CD8+ T cells in mouse strains expressing both H-2k

and T cell receptor V alpha a haplotypes: determination with a V alpha 2-specific monoclonal antibody.. Eur J Immunol. 1992; 22 (2):399-404.
In Supplementary Figures 3b,c, DN thymocytes from P14 transgenic mice were positively stained with this antibody.

PE-conjugated anti-mCD8α (clone 53-6.7), BD Pharmingen, Catalog No. 553033
Bierer BE, Sleckman BP, Ratnofsky SE, Burakoff SJ. The biologic roles of CD2, CD4, and CD8 in T-cell activation. Annu Rev Immunol. 1989; 7:579-599.
Fujiura Y, Kawaguchi M, Kondo Y, et al. Development of CD8 alpha alpha+ intestinal intraepithelial T cells in beta 2-microglobulin- and/or TAP1-deficient mice. J Immunol. 1996; 156(8):2710-2715.
In Figure 5b DN thymocytes from P14 transgenic mice re-expressing CD8 were positively stained with this antibody.

PE-conjugated isotype RatIgG2α,λ (clone B39-4), BD Pharmingen, Catalog No. 557076
Suggested isotype control antibody for anti-mTCR Vα2 (BD Catalog No. 553289)
In Supplementary Figures 3b,c, DN thymocytes from P14 transgenic mice were negatively stained with this antibody.

PE-conjugated isotype RatIgG2α,κ (clone A95-1), BD Pharmingen, Catalog No. 553989
Suggested isotype control antibody for anti-mCD8α (BD Catalog No. 553033)
Bianchi, Maria, et al. "Susceptibility of primary human airway epithelial cells to Bordetella pertussis adenylate cyclase toxin in two- and three-dimensional culture conditions." Innate Immunity (2020): 1753425920979354
Tromp, Angelino T., et al. "Pre-existing antibody-mediated adverse effects prevent the clinical development of a bacterial anti-inflammatory protein." Disease models & mechanisms 13.9 (2020).
In Figures 5b, DN thymocytes from P14 transgenic mice were negatively stained with this antibody.

PE-conjugated isotype american hamster IgG2,κ (clone B81-3), BD Pharmingen, Catalog No. 550085
Mendrick DL, Kelly DM. Temporal expression of VLA-2 and modulation of its ligand specificity by rat glomerular epithelial cells in vitro. Lab Invest. 1993; 69(6):690-702.
In Supplementary Figures 3b,c, DN thymocytes from P14 transgenic mice expressing PD-1 were negatively stained with this antibody.

PE-conjugated anti-mPD-L1 (clone MIH5), ThermoFisher Scientific, Catalog No. 12-5982-82
Lacotte, Stéphanie, et al. "Impact of myeloid-derived suppressor cell on Kupffer cells from mouse livers with hepatocellular carcinoma." Oncoimmunology 5.11 (2016): e1234565.
Vo, Manh-Cuong, et al. "Lenalidomide and programmed death-1 blockade synergistically enhances the effects of dendritic cell vaccination in a model of murine myeloma." Frontiers in immunology 9 (2018): 1370.

biotinylated anti-human IgG1 (clone HP6070), ThermoFisher Scientific, Catalog No. MH1515
Vendel, Andrew C., et al. "B and T lymphocyte attenuator regulates B cell receptor signaling by targeting Syk and BLNK." The Journal of Immunology 182.3 (2009): 1509-1517.
Gonzalez, Lino C., et al. "A coreceptor interaction between the CD28 and TNF receptor family members B and T lymphocyte attenuator and herpesvirus entry mediator." Proceedings of the National Academy of Sciences 102.4 (2005): 1116-1121.

BV421-conjugated anti-CD4 (clone GK1.5), Biolegend, Catalog No. 100438
Cignarella F, Cantoni C, Ghezzi L, Salter A, Dorsett Y, Chen L, Phillips D, Weinstock GM, Fontana L, Cross AH, Zhou Y, Piccio L. Intermittent Fasting Confers Protection in CNS Autoimmunity by Altering the Gut Microbiota. Cell Metab. 2018 Jun 5;27 (6):1222-1235.
Xu J, Mathur J, Vessières E, Hammack S, Nonomura K, Favre J, Grimaud L, Petrus M, Francisco A, Li J, Lee V, Xiang FL, Mainquist JK, Cahalan SM, Orth AP, Walker JR, Ma S, Lukacs V, Bordone L, Bandell M, Laffitte B, Xu Y, Chien S, Henrion D, Patapoutian A. GPR68 Senses Flow and Is Essential for Vascular Physiology. Cell. 2018 Apr 19;173(3):762-775.
In Figure 5a thymocytes from P14 transgenic mice were positively stained with this antibody.

PE-conjugated anti-H2-Db (clone KH95), Biolegend, Catalog No. 111508
Wolf Y, Bartok O, Patkar S, Eli GB, Cohen S, Litchfield K, Levy R, Jiménez-Sánchez A, Trabish S, Lee JS, Karathia H, Barnea E, Day CP, Cinnamon E, Stein I, Solomon A, Bitton L, Pérez-Guijarro E, Dubovik T, Shen-Orr SS, Miller ML, Merlino G, Levin Y, Pikarsky E, Eisenbach L, Admon A, Swanton C, Ruppin E, Samuels Y. UVB-Induced Tumor Heterogeneity Diminishes Immune Response in Melanoma. Cell. 2019 Sep 19;179(1):219-235.e21.
Ye X, Waite JC, Dhanik A, Gupta N, Zhong M, Adler C, Malahias E, Ni M, Wei Y, Gurer C, Zhang W, Macdonald LE, Murphy AJ, Sleeman MA, Skokos D. Endogenous retroviral proteins provide an immunodominant but not requisite antigen in a murine immunotherapy tumor model. Oncoimmunology. 2020 May 13;9(1):1758602.

PE-conjugated anti-mCD8α (clone 53-6.7), Biolegend, Catalog No. 100708
Xu C, Ruan B, Jiang Y, Xue T, Wang Z, Lu H, Wei M, Wang S, Ye Z, Zhai D, Wang L, Lu Z. Antibiotics-induced gut microbiota dysbiosis promotes tumor initiation via affecting APC-Th1 development in mice. Biochem Biophys Res Commun. 2017 Jun 24;488(2):418-424.
Manrique-Rincón AJ, Beraldo CM, Toscaro JM, Bajgelman MC. Exploring Synergy in Combinations of Tumor-Derived Vaccines That Harbor 4-1BBL, OX40L, and GM-CSF. Front Immunol. 2017 Sep 19;8:1150.
In Figure 5a thymocytes from P14 transgenic mice were positively stained with this antibody.

PE-conjugated isotype ratIgG1,κ (clone RTK2071), Biolegend, Catalog No. 400408
Nam GH, Lee EJ, Kim YK, Hong Y, Choi Y, Ryu MJ, Woo J, Cho Y, Ahn DJ, Yang Y, Kwon IC, Park SY, Kim IS. Combined Rho-kinase inhibition and immunogenic cell death triggers and propagates immunity against cancer. Nat Commun. 2018 Jun 4;9(1):2165.
Wu L, Liu H, Guo H, Wu Q, Yu S, Qin Y, Wang G, Wu Q, Zhang R, Wang L, Zhang L, Liu C, Jiao S, Liu T. Circulating and tumor-infiltrating myeloid-derived suppressor cells in cervical carcinoma patients. Oncol Lett. 2018 Jun;15(6):9507-9515.

APC-conjugated anti-CD44 (clone IM7) was from Tonbo biosciences. Catalog No. 20-0441-U100
Hao Shi, Nicole M. Chapman, Jing Wen, Cliff Guy, Lingyun Long, Yogesh Dhungana, Sherri Rankin, Stephane Pelletier, Peter Vogel, Hong Wang, Junmin Peng, Kun-Liang Guan, and Hongbo Chi. Amino Acids License Kinase mTORC1 Activity and Treg Cell Function via Small G Proteins Rag and Rheb. Immunity. 2019 Dec 17;51(6):1012-1027.e7.
Oh H, Grinberg-Bleyer Y, Liao W, Maloney D, Wang P, Wu Z, Wang J, Bhatt DM, Heise N, Schmid RM, Hayden MS, Klein U, Rabadan R and Ghosh S. 2017. An NF-κB Transcription-Factor-Dependent Lineage-Specific Transcriptional Program Promotes Regulatory T Cell Identity and Function. Immunity.
In Supplementary Figure 3a, DN thymocytes from P14 transgenic mice were positively stained with this antibody.

Biotinylated anti-His tag (cat. 34440) from Qiagen.
Reinemann DN, Sturgill EG, Das DK, Degen MS, Vörös Z, Hwang W, Ohi R, Lang MJ. Collective Force Regulation in Anti-parallel Microtubule Gliding by Dimeric Kif15 Kinesin Motors. Curr Biol. 2017 Sep 25;27(18):2810-2820.
Reinemann DN, Norris SR, Ohi R, Lang MJ. Processive Kinesin-14 HSET Exhibits Directional Flexibility Depending on Motor Traffic. Curr Biol. 2018 Jul 23;28(14):2356-2362.

Eukaryotic cell lines

Policy information about cell lines

Cell line source(s)	293T and CHO-K1 cells from ATCC
Authentication	Authentication was not performed.
Mycoplasma contamination	Mycoplasma contamination was not tested.
Commonly misidentified lines (See ICLAC register)	No commonly misidentified cell lines were used in the study.

Animals and other organisms

Policy information about studies involving animals; ARRIVE guidelines recommended for reporting animal research

Laboratory animals	6-7-week-old female P14 (LCMV-gp33 specific transgenic TCR) mouse (C57BL/6J background) were housed under the following conditions: Daylight cycle 7am - 9pm; Darklight cycle 9pm - 7am; Ambient Temperature 72 F; Humidity 30-70%.
Wild animals	No wild animals were used in this study.
Field-collected samples	This study did no involve data collected from the field.
Ethics oversight	P14 transgenic, PDCD1-/- P14 transgenic, and C57BL/6 mice were housed at the Emory University Department of Animal Resources facility and used in accordance with National Institutes of Health and the Emory University Institutional Animal Care and Use Committee guidelines.

Note that full information on the approval of the study protocol must also be provided in the manuscript.

Human research participants

Policy information about studies involving human research participants

Population characteristics	Healthy male and female (not pregnant) adult donors who weigh at least 110 pounds. Human RBCs were used in this study only as a tool to present biotinylated ligands. The property or biology of RBCs is not the focus of this study.
Recruitment	Participants were recruitment from students and staffs on campus. Participant volunteered for blood donation with full consent. There was no self-selection bias.
Ethics oversight	Institutional Review Board of the Georgia Institute of Technology

Note that full information on the approval of the study protocol must also be provided in the manuscript.

Flow Cytometry

Plots

Confirm that:

- [x] The axis labels state the marker and fluorochrome used (e.g. CD4-FITC).
- [x] The axis scales are clearly visible. Include numbers along axes only for bottom left plot of group (a 'group' is an analysis of identical markers).
- [x] All plots are contour plots with outliers or pseudocolor plots.
- [x] A numerical value for number of cells or percentage (with statistics) is provided.

Methodology

Sample preparation	Thymocytes were collected from the thymus of P14 mice. Antibodies used for flow sorting were CD4 (GK1.5;Biolegend), CD8 (53-6.7;Biolegend). Staining was performed by incubating antibodies with cells at a concentration of 2x10^7 cells/ml in staining buffer (PBS with 2%FBS) for 30 min on ice, followed by three washes with staining buffer. Activated T cells and RBC were stained following the same protocol with corresponding antibodies.
Instrument	BD FACS Aria, LSRII, Fortessa, Accuri
Software	Flowjo V10
Cell population abundance	DN population was 3-7% of thymocytes. 2-3X10^5 DN cells/thymus were sorted with 95-97% of purity.
Gating strategy	Lymphocytes were gated on FSC/SSC. Gating were based on positive vs negative populations of the corresponding staining.

☒ Tick this box to confirm that a figure exemplifying the gating strategy is provided in the Supplementary Information.
